# Development of Shortened miR-506-3p Mimics Exhibiting Strong Differentiation-Inducing Activity in Neuroblastoma Cells

**DOI:** 10.3390/molecules28176295

**Published:** 2023-08-28

**Authors:** Nakya Mesa-Diaz, Mitchell T. Smith, Daniela F. Cardus, Liqin Du

**Affiliations:** Department of Chemistry and Biochemistry, Texas State University, San Marcos, TX 78666, USA; nakyadiaz@gmail.com (N.M.-D.); smithm2152@gmail.com (M.T.S.); dfc32@txstate.edu (D.F.C.)

**Keywords:** miR-506-3p, neuroblastoma, mimics, differentiation, analog, truncation

## Abstract

microRNA mimics are synthetic RNA molecules that imitate the mature miRNA duplexes and their functions. These mimics have shown promise in treating cancers. Nucleotide chemical modifications of microRNA mimics have been investigated and have improved the stability of miRNA mimics. However, the potential therapeutic benefit of mimic analogs based on sequence modifications has not been explored. miR-506-3p was identified as a differentiation-inducing microRNA in neuroblastoma cells, suggesting the potential of applying the miR-506-3p mimic in neuroblastoma differentiation therapy. In this study, we explored the possibility of developing shortened miR-506-3p analogs that can maintain differentiation-inducing activities comparable to the wild-type miR-506-3p mimic. We found that deleting up to two nucleotides at either the 3′ end or within the middle region of the miR-506-3p sequence fully maintained the differentiation-inducing activity when compared to the wild-type mimic. Deleting up to four nucleotides from the 3′ end or deleting three nucleotides in the middle positions diminished the differentiation-inducing activity, but the analogs still maintained differentiation-inducing activities that were significantly higher than the negative control oligo. The shortened analog designs potentially benefit patients from two perspectives: (1) the reduced cost of manufacturing shortened analogs, and (2) the reduced non-specific toxicity due to their smaller molecular sizes.

## 1. Introduction

Neuroblastoma is one of the most aggressive types of pediatric cancer. It accounts for about 15% of cancer-related childhood deaths [1,2]. Neuroblastoma originates from neural crest precursor cells that fail to complete the differentiation process [2,3]. This tumorigenic mechanism sets the foundation for differentiation therapy, an approach to induce neuroblastoma cells into terminal differentiation and thereby arrest cancer cell growth. Currently, the differentiation agent 13-*cis*-retinoic acid (RA) is used as the standard of care for post-remission maintenance therapy in high-risk neuroblastoma patients [2]. Such treatment only modestly improves patient viability. More than 50% of the RA-treated patients still develop recurrence [4,5], indicating that a significant number of high-risk neuroblastoma patients either do not respond to RA or develop resistance to RA post-treatment. Differentiation-inducing agents that are mechanistically distinct from RA are needed for these patients.

Synthetic RNA oligonucleotides (oligos) that mimic or antagonize endogenous microRNA (miRNAs) functions represent a unique class of biomolecules for developing innovative cancer therapies, including differentiation therapies for neuroblastoma. miRNAs play a critical role in tumorigenesis by functioning as either oncogenes or tumor suppressor genes [6,7,8,9]. In miRNA replacement therapy based on tumor-suppressive miRNA mimics, synthetic oligos used to raise intracellular miRNA levels have been investigated as anti-cancer agents and have shown great therapeutic promise [10,11]. miRNAs that have strong differentiation-inducing activities in neuroblastoma cells have been identified [12,13,14,15,16], suggesting the potential of applying miRNA mimics to neuroblastoma differentiation therapy. Due to the rapid advances in technologies for in vivo delivery of nucleic acids, nucleic-acid-based therapeutics have become increasingly feasible [17,18]. Pre-clinical and clinical investigations have been conducted for various types of nucleic acid drugs, including small inhibitory RNAs (siRNAs) [19,20], miRNAs [17,18], and other therapeutic nucleic acids [21,22,23]. These nucleic acid drugs have been proven to be effective in treating various human diseases including cancer. For therapeutic miRNA mimics, nucleotide chemical modifications have also been extensively investigated to improve their stability and achieve sustained intracellular activity [24,25,26]. However, modifications of nucleotide sequences of miRNA mimics aimed at reducing the molecular sizes of the mimics (i.e., shortening the miRNA mimic in length) have not been explored, which leaves a knowledge gap in the development of miRNA-mimic-based therapeutics. Filling this gap may facilitate the designing of more effective therapeutic mimics.

The lengths of endogenously expressed mature miRNAs typically vary between 18 and 24 nucleotides [27]. Although it has been well established that the seed sequence region (minimum seed sequence is nucleotides 2–6 counting from the 5′ end) located at the 5′ end of a miRNA is responsible for defining the targetome of the miRNA [28]. The functions of the non-seed sequence regions have not been fully characterized. Several studies have suggested that nucleotides at certain positions of the non-seed regions are either not required for the target site recognition or not involved in direct miRNA–target interactions. For example, studies have shown that the first nucleotide at the 5′ end of the mRNA is not required to form a base pair with its target site [28,29]. Regarding the function of the 3′ end non-seed sequence region, Pratt et al., preliminarily characterized its role when studying the RNA-induced silencing complex (RISC)-directed guide oligo interaction with its target strand [29]. They found that the nucleotides at positions 11–18 disordered in the crystal structure of the target–guide–Argonaute (Ago) interaction complex. This finding suggests that at least a portion of the 3′ non-seed region of the miRNA maintains a high degree of flexibility, and interactions of this region with the target sequence are not required for miRNA function. The above studies altogether suggest that deleting a certain number of nucleotides at the non-seed sequence region of a miRNA may be tolerable for maintaining its biological function. This inspired us to explore shortened mimic designs for miR-506-3p, a differentiation-inducing miRNA that was identified by our research group. We discovered multiple shortened miR-506-3p mimic analogs with their differentiation-inducing activity comparable to that of the full-length WT miR-506-3p mimic. A therapeutic miRNA mimic with reduced molecular size would have at least two advantages over the full-length miRNA mimic: (1) it reduces the cost of synthesizing the mimics, and (2) it potentially reduces the non-specific side effects on normal cells (e.g., non-specific cytotoxicity) due to the reduced sizes. These advantages would be beneficial to patients when miR-506-3p mimics are translated into clinical applications for treating neuroblastoma patients. 

## 2. Results

### 2.1. Dose-Dependent Differentiation-Inducing Activity of Wild-Type (WT) miR-506-3p Determined in BE(2)-C Cells

**Figure 1:**
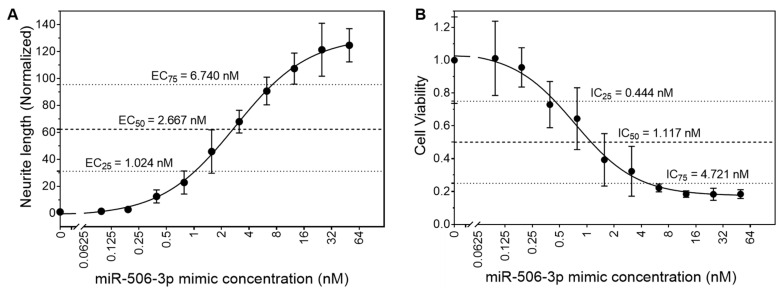
Dose-dependent effect of WT miR-506-3p on neurite outgrowth and cell viability in BE(2)-C cells. Cells were transfected with a series of concentrations of the WT miR-506-3p mimic and cultured for 4 days. Neurite outgrowth and cell viability were then measured, and the dose-dependent responses were analyzed as described in Section 4. (**A**) Dose-dependent effect of the WT miR-506-3p mimic on neurite outgrowth. Shown are the average value of normalized neurite length (black dot) with its corresponding standard deviation bar generated from three replicates for each concentration. (**B**) Dose-dependent effect of the WT miR-506-3p mimic on cell viability. Shown are the average value of normalized cell viability (black dot) with its corresponding standard deviation bar generated from three replicates for each concentration. For both (**A**) and (**B**), the white squares that are used to break the x-Axes and fitted curves signify that the 0 nM concentration (located on the left side of the white square) cannot be log2 transformed, whereas all other concentrations used in the experiments (located on the right side of the white square) are log2 transformed for the purpose of generating the dose-dependent curves.

In order to determine the appropriate concentrations to compare the differentiation-inducing activities of miR-506-3p analogs, we first measured the dose-dependent effect of the WT miR-506-3p mimic on neurite outgrowth in the neuroblastoma cell line BE(2)-C. As shown in Figure 1A, WT miR-506-3p shows a dose-dependent effect on neurite outgrowth with an EC_50_ value of 2.667 nM. We then measured the effect of the WT miR-506-3p mimic on the number of viable cells (i.e., the cell viability as presented in the figures) using MTT assay, which reflects the effect of WT miR-506-3p mimic on the cell proliferation capacity. Figure 1B shows that WT miR-506-3p significantly reduces cell viability in a dose-dependent manner, with the IC_50_ value being 1.117 nM, which indicates that the neurite outgrowth induced by the miRNA mimic is accompanied by cell growth arrest. The cell growth arrest accompanied by the neurite outgrowth induced by miR-506-3p demonstrates that cell differentiation was truly induced by this miRNA. The ranges of EC_25_–EC_75_ (1.024–6.740 nM) and IC_25_–IC_75_ (0.444–4.721 nM) largely overlap, which suggests that the induction of neurite outgrowth is tightly correlated with cell growth arrest. The data above helped us to determine the two concentrations (i.e., 2 nM and 5 nM), which are within the linear region of the dose-dependent responses and allow for accurate comparison of the activities of the WT mimic and analogs, used to investigate the differentiation-inducing activities of miR-506-3p analogs.

### 2.2. Multiple 3′-End-Truncated miR-506-3p Analogs Show Strong Differentiation-Inducing Activities in Neuroblastoma Cell Line BE(2)-C

As shown in Table 1, we initially designed six shortened analogs of miR-506-3p by making deletions from the 3′ end (506-TRN1 to 506-TRN6). The negative control oligos were designed by making point mutations in the seed sequence. As additional controls, we also made two shortened analogs by deleting up to two nucleotides from the 5′ end (506-5′TRN1 and 506-5′TRN2) and one shortened analog by deleting one nucleotide from each end (506-TRN1-5′TRN1). A carrier strand for each analog was constructed for generating double-stranded oligonucleotides.

**Figure 2:**
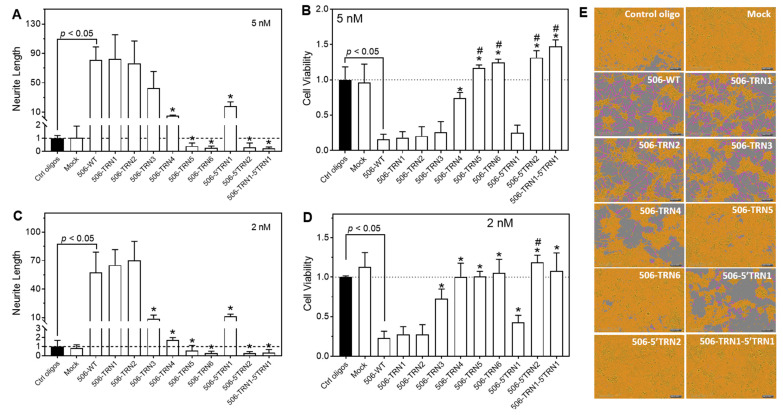
Effect of 3′-end-truncated miR-506-3p analogs on neurite outgrowth and cell viability in BE(2)-C cells. Cells were transfected with the control oligos, the WT miR-506-3p mimic (WT-506), or the indicated 9 miR-506-3p analogs at final concentrations of 2 nM and 5 nM for 4 days. Mock treatment represents cells treated with only transfection reagents. Neurite outgrowth and cell viability were measured as above. (**A**,**C**) Effect of the WT mimic and truncated miR-506-3p analogs on neurite outgrowth. * significant decrease (*p* < 0.05) in neurite length compared to 506-WT. (**B**,**D**) Effect of the WT mimic and analogs on cell viability. * significant increase (*p* < 0.05) in cell viability compared to 506-WT. # significant increase (*p* < 0.05) in cell viability compared to control oligos. (**E**) Representative cell images, with cell bodies marked in yellow and neurites in pink, for the indicated treatments. Dot lines in (**A**–**D**) signify the values of the control groups.

We examined the differentiation-inducing activity of these oligos by measuring their effect on neurite outgrowth and cell growth arrest. As shown in Figure 2, deleting up to two nucleotides (506-TRN1 and 506-TRN2) from the 3′ end did not impair the differentiation-inducing activity compared to the WT mimic at both 5 nM and 2 nM concentrations, as measured by neurite outgrowth (Figure 2A,C) and cell viability (Figure 2B,D). Deleting three nucleotides (506-TRN3) from the 3′ end did not significantly impair the differentiation-inducing activity at the 5 nM concentration. At the 2 nM concentration, however, its neurite-inducing activity was modestly but significantly reduced compared to the WT mimic, whereas its effect on cell viability was not significantly different from the WT mimic. By contrast, deleting four or more nucleotides from the 3′ end either dramatically decreased or totally abolished the differentiation-inducing activity. Overall, our results indicate that the activity of miR-506-3p mimics can be maintained after removing two nucleotides from the 3′ end of the WT miR-506-3p sequence, whereas deleting three to six nucleotides showed a gradually increased impairment of the differentiation-inducing activity, albeit with deleting three nucleotides still showing satisfactory activity.

As expected, deleting two nucleotides from the seed-sequence end (i.e., 5′ end) (506-5′TRN2) completely eradicated the differentiation-inducing activity. By contrast, deleting one nucleotide from the 5′ end (506-5′TRN1) dramatically but not fully diminished the differentiation-inducing activity; this finding is not surprising because it is consistent with previous publications, which reported that the base pairings of the first nucleotide at the 5′ end of a miRNA with their target sites are not always required for repressing the expression of its target genes [28]. Interestingly and unexpectedly, however, a one-nucleotide deletion at each end (506-TRN1-5′TRN1) completely eradicated the differentiation-inducing activity to the level of 506-5′TRN2, even though deleting only one nucleotide from the 3′ end (506-TRN1) had no impact on the differentiation-inducing activity.

### 2.3. Deleting Two Nucleotides in the Middle Positions of the miR-506-3p Sequence Did Not Impair the Differentiation-Inducing Activity

**Figure 3:**
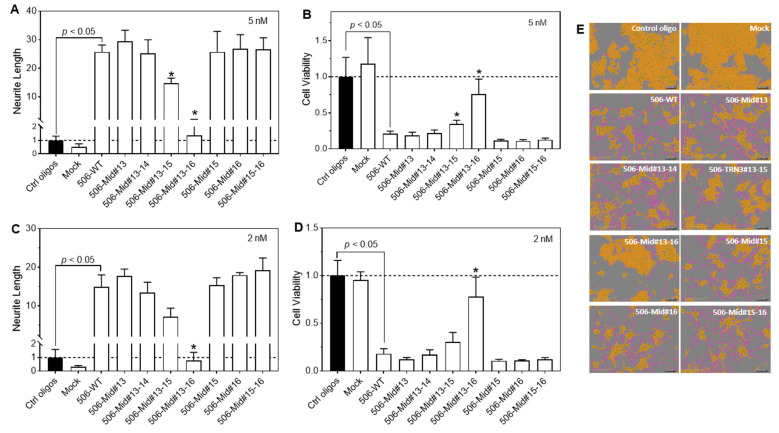
Effect of miR-506-3p analogs shortened in the middle positions on neurite outgrowth and cell viability in BE(2)-C cells. Cells were transfected with control oligos, WT-506, or the indicated 7 miR-506-3p analogs at final concentrations of 2 nM and 5 nM for 4 days. Neurite outgrowth and cell viability were measured as above. (**A**,**C**) Effect of the WT mimic and analogs on neurite outgrowth. (**B**,**D**) Effect of the WT mimic and analogs on cell viability. * *p* < 0.05 compared to 506-WT. (**E**) Representative cell images, with cell bodies marked in yellow and neurites in pink, for the indicated treatments. Dot lines in (**A**–**D**) signify the values of the control groups.

To further explore alternative designs of shortened miR-506-3p mimics, we made several deletions in the middle, non-seed sequence positions of miR-506-3p (Table 2). As seen in Figure 3A–E, deleting up to two nucleotides within the nucleotide positions #13–#16 (analogs 506-Mid#13, 506-Mid#13-14, 506-Mid#15, 506-Mid#16, and 506-Mid#15-16) maintained a differentiation-inducing activity equal to that of the WT miR-506-3p mimic. Deleting three nucleotides (506-Mid#13-15) still maintained a satisfactory differentiation-inducing activity, with its effect on neurite outgrowth and cell viability only modestly reduced compared to the WT mimic. By contrast, deleting four nucleotides (506-Mid#13-16) in this region almost abolished the differentiation-inducing activity.

### 2.4. Effect of Combined 3′-End and Middle-Position Deletions on the Differentiation-Inducing Activity

**Figure 4:**
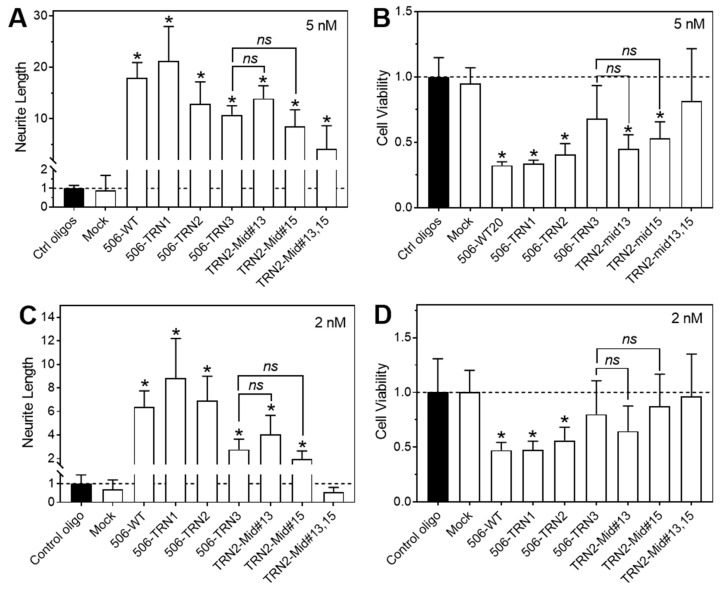
Effect of combined 3′-end and mid-position deletions on the activity of the miR-506-3p mimic in BE(2)-C cells. Cells were transfected with control oligos, WT-506, or the indicated 6 miR-506-3p analogs at final concentrations of 2 nM and 5 nM for 4 days. Neurite outgrowth and cell viability were measured as above. (**A**,**C**) Effect of the WT mimic and analogs on neurite outgrowth. (**B**,**D**) Effect of the WT mimic and analogs on cell viability. *ns*, not significant (*p* > 0.05); * *p* < 0.05 compared to the control oligos. Dot lines in the figures signify the values of the control groups.

Encouraged by the findings from the 3′-end and middle-position deletions, we intended to investigate analogs that combine 3′-end truncations and middle-position deletions (sequences listed in Table 3). As shown in Figure 4, combined deletions of three nucleotides (TRN2-Mid#13 and TRN2-Mid#15) did not show improved differentiation-inducing activity compared to the three-nucleotide deletion at the 3′ end. Combined deletions of four nucleotides (TRN2-Mid#13,15) completely abolished the differentiation-inducing activity at 2 nM, which is consistent with what was observed with four-nucleotide 3′-end truncation (506-TRN4, Figure 2). Taken together, our results suggest that a minimum length of nucleotides in the non-seed region is required for maintaining the differentiation activity of the miR-506-3p analogs, with shortening the non-seed region by four nucleotides dramatically impairing or abolishing the differentiation-inducing activity.

### 2.5. Effect of 5′ U Addition to the Truncated Analogs on Their Differentiation-Inducing Activities

**Figure 5:**
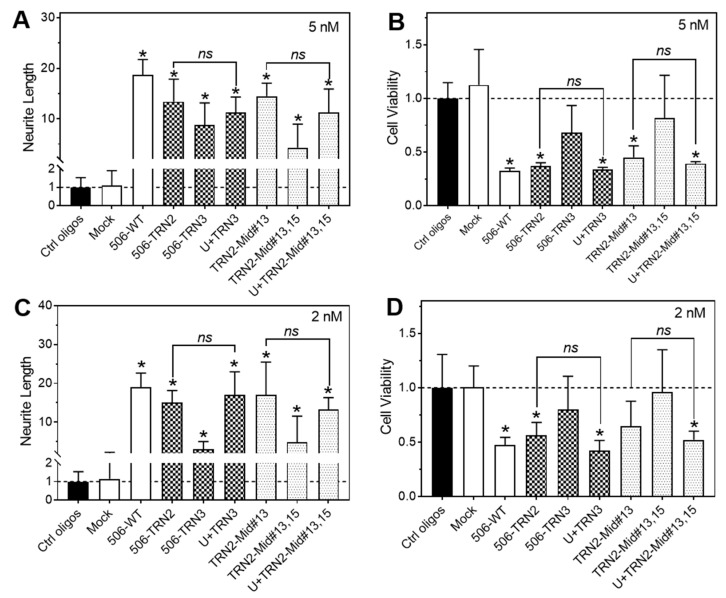
Effect of 5′ U additions on the activity of miR-506-3p analogs in BE(2)-C cells. Cells were transfected with control oligos, WT-506, or the indicated 5 miR-506-3p analogs at final concentrations of 2 nM and 5 nM for 4 days. Neurite outgrowth and cell viability were measured as above. (**A**,**C**) Effect of the WT mimic and analogs on neurite outgrowth. (**B**,**D**) Effect of the WT mimic and analogs on cell viability. *ns*, not significant (*p* > 0.05); * *p* < 0.05 compared to the control oligos. Dot lines in the figures signify the values of the control groups.

miR-506-3p shares the same seed sequence with miR-124-3p, and they have comparable differentiation-inducing activities in neuroblastoma cells [16]. An isoform of miR-124-3p (miR-124-3p.2) has an extra uracil (U) at the 5′ end compared to miR-506-3p, with its sequence being UUAAGGCACGCGGUGAAUGC [30,31]. In addition, it has been suggested that some Ago proteins have preferences for 5′ U when the Ago protein recruits the guide RNA into the RISC complex on the target site [29,32]. This motivated us to investigate whether adding a 5′ U to the truncated analogs would enhance the differentiation-inducing activity (sequences listed in Table 3). As shown in Figure 5, adding a 5′ U to the 506-TRN3 analog (i.e., U+TRN3) restored its differentiation-inducing activity to that of 506-TRN2, which is the same length as U+TRN3. However, the differentiation-inducing activity of U+TRN3 was not significantly higher than that of 506-TRN2. Likewise, U+TNR2-Mid#13,15 did not show a significantly enhanced differentiation-inducing activity compared to TNR2-Mid#13. These results altogether indicate that adding a 5′ U is not beneficial for designing truncated miR-506-3p mimics. 

### 2.6. Shortened miR-506-3p Analogs Exhibit Differentiation-Inducing Activity in Multiple Neuroblastoma Cell Lines

**Figure 6:**
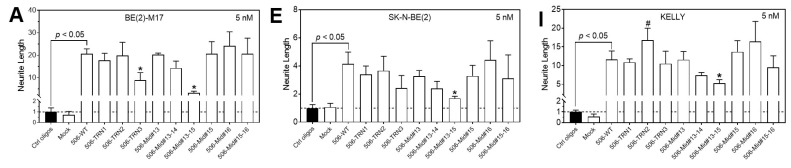

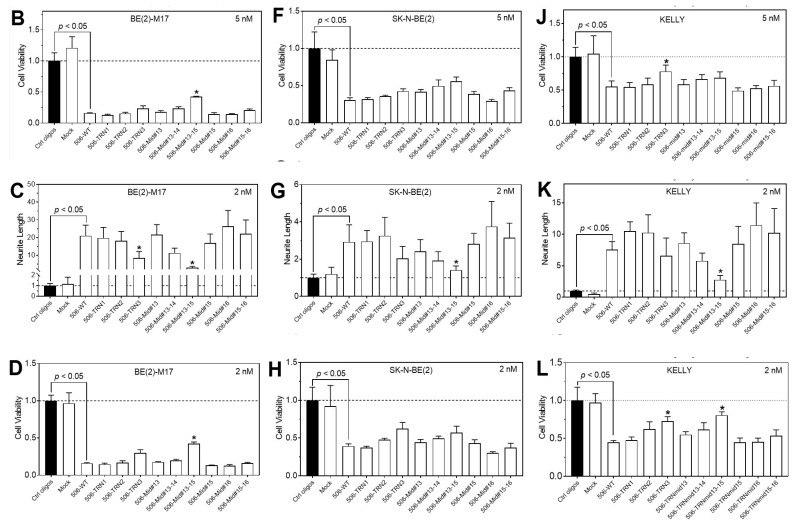
Effect of miR-506-3p analogs on neurite outgrowth and cell viability in multiple neuroblastoma cell lines. Cells were transfected with control oligos, WT-506, or the indicated 9 miR-506-3p analogs at final concentrations of 2 nM and 5 nM for 4 days. Neurite outgrowth and cell viability were measured as above. Shown are the neurite outgrowth and cell viability analyses for BE(2)-M17 (**A**–**D**), SK-N-BE(2) (**E**–**H**), and KELLY cells (**I**–**L**). * significantly reduced activity compared to 506-WT (*p* < 0.05); *#* significantly increased activity compared to 506-WT (*p* < 0.05). Dot lines in the figures signify the values of the control groups.

From the above investigations, we identified multiple shortened analogs with satisfactory differentiation-inducing activities in BE(2)-C cells. To determine whether these analogs have generic activities in neuroblastoma cells, we tested nine analogs in additional neuroblastoma cell lines. As shown in Figure 6, the nine analogs were active in all three cell lines, BE(2)-M17 (Figure 6A–D), SK-N-BE(2) (Figure 6E–H), and KELLY (Figure 6I–L) cells, and their activities were comparable to those observed in BE(2)-C cells. These results suggest that our identified analogs have a generic differentiation-inducing effect in neuroblastoma cells.

### 2.7. Shortened miR-506-3p Analogs Exhibit Potent Activity in Arresting the Long-Term Proliferation of Neuroblastoma Cells

**Figure 7:**
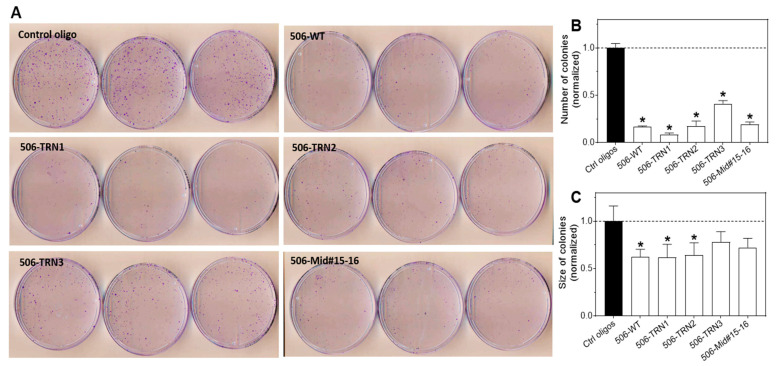
Effect of miR-506-3p analogs on colony formation. Colony formation assay as a function of the WT miR-506-3p mimic and analogs was examined in BE(2)-C cells. Cells were transfected with 20 nM of the control oligos, WT-506, or the indicated 5 miR-506-3p analogs, and colony formation was examined as described in the Materials and Methods. Shown are plate images of the colony formation assay (**A**) and quantified colony numbers (**B**) and sizes (**C**). * *p* < 0.05 compared to control. Dot lines in (**B**,**C**) signify the values of the control groups.

To examine the long-term capacity of the shortened analogs to repress cell proliferation, we investigated the effect of selected analogs on colony formation in BE(2)-C cells. As shown in Figure 7, all four analogs (506-TRN1, 506-TRN2, 506-TRN3, and 506-mid#15-16) significantly reduced the number of colonies compared to negative control oligos (Figure 7A,B), although the decreases in the colony sizes (Figure 7C) were not as dramatic when compared to the negative control oligo. These results indicate that the shortened analogs effectively repress the long-term proliferation of neuroblastoma cells.

## 3. Discussion

In this study, we explored the possibility of developing shortened miR-506-3p mimics as potential therapeutic agents, which have not been reported previously. We identified a group of shortened miR-506-3p mimic analogs that maintain strong differentiation-inducing activities in neuroblastoma cells. This group of miR-506-3p mimic analogs with reduced molecular mass will benefit the future development of miR-506-3p-mimic-based therapeutic agents if miR-506-3p mimics are successfully translated into neuroblastoma therapy. In addition, knowledge gained from our study may provide guidance for developing shortened miRNA mimic analogs of other miRNAs that have therapeutic potential. One benefit of reducing the length of the miRNA mimic is to reduce the cost of synthesizing the miRNA mimic. Another potential benefit is to decrease the off-target effects due to the reduced binding affinity of miRNA with the non-specific binding sites. It has been demonstrated that the target specificity of a miRNA is determined by its seed sequence, the nucleotides 1–8 counting from the 5′ end of the miRNA sequence. Therefore, shortening in the non-seed sequence will not increase the off-target effect. On the contrary, it has been indicated that the preferential pairing at the non-seed region is important to increase the miRNA–target hybrid stability [33,34,35]. It is possible that the non-seed region binds to a certain off-target region and leads to an off-target effect. Therefore, shortening the non-seed region potentially reduces the spectrum of mRNAs that are targeted by the non-seed region of the shortened analogs, thereby potentially reducing the off-target effect of the miRNA.

We demonstrated that deleting up to two nucleotides either at the 3′ end or within the middle nucleotide positions 13–16 of the miR-506-3p sequence fully maintained the differentiation-inducing activity of the mimics. This non-selective tolerance of two-nucleotide deletions at both the 3′ end and the middle positions of the 3′ non-seed region suggests that a minimum length of the non-seed region rather than the specific nucleotide sequence in this region is critical for the interaction of miR-506-3p with its RISC-associated target sites. While the tolerance for the 3′-end deletion cannot be explained by the current literature, the mechanism underlying the tolerance for the middle-region deletion can be, at least partially, explained by the work of Pratt et al. [29]. In investigating the RISC-directed guide oligo interaction with its target strand, Pratt et al., found that nucleotides 11–18 of the guide oligo seemed to be largely disordered in the crystal structure of the target–guide–Ago interaction complex [29]. This finding suggests that this region of the miRNA maintains a high degree of structural variability in the RISC complex, and that the base pairing of this region with the target sequence is not required for miRNA function. This explains why deleting up to two nucleotides between nucleotides 13 and 16 did not impair the differentiation-inducing activity of the miR-506-3p mimic. Contrary to the above findings, however, several published works have shown that the preferential pairing at the nucleotides 13–16 region is important in increasing the miRNA–target hybrid stability, and thereby may lead to enhanced target down-regulation [33,34,35]. Therefore, the requirement for base pairing in nucleotides 13–16 might be miRNA-specific, so that some miRNAs require binding to their targets at nucleotides 13–16 whereas other miRNAs do not. For each specific therapeutic miRNA mimic, the positions in the non-seed regions that tolerate deletions certainly need to be individually investigated when developing shortened mimics.

It is worth noting that deleting three nucleotides from either the 3′ end or the middle position will still maintain a significantly higher differentiation-inducing activity when compared to the negative control in all four cell lines. Their activities are especially satisfactory in BE(2)-M17, SK-N-BE(2), and KELLY cells, with their activities only decreasing modestly at both concentrations compared to the WT miR-506-3p mimic. From the perspective of therapeutic potential, the three-nucleotide shortened analog designs with slightly reduced differentiation-inducing activity might still be beneficial—shortening one more nucleotide might be able to effectively antagonize the increased risk of side effects associated with a two-nucleotide-shortened analog while still maintaining satisfactory therapeutic efficacy. Their therapeutic efficacies and toxicities certainly need to be investigated and compared in in vivo studies.

In contrast to the tolerance for two-nucleotide deletions at the 3′ non-seed region, deleting only one nucleotide from the 5′ end dramatically impaired but did not completely abolish the differentiation-inducing activity. These results can be explained by findings from many previous studies [28,29,32]. It is known that the eight nucleotides at the 5′ end of mature miRNAs are the seed region that is responsible for target site recognition [28]. However, the binding patterns of the miRNA seed regions with their target sites vary depending on the sequences of both the miRNA and their targets. The core seed sequence required for forming base pairs with the target site comprises nucleotides 2–6 [28], allowing one or two nucleotides at the 5′ or the 3′ end of the 1–8 seed region unpaired with the target sites. Among these variations, non-base pairing in the first nucleotide seems to be more common than any other variation, which supports that the first nucleotide does not play a critical role in target recognition, at least in most cases [28]. It has been speculated that the first nucleotide may have a role other than target recognition. Indeed, in studying the crystal structure of the RNA-induced silencing complex (RISC), Pratt et al., found that, when the Ago protein binds to the guide oligonucleotide (oligo) and recruits the oligo into the RISC on the target site, the primary role of the first nucleotide at the 5′ end of the small RNA was to fit into a small pocket of the protein complex [29]. Our finding suggests that impaired binding of the first nucleotide with the RISC complex only partially disrupted the miRNA–target interaction complex, providing a more quantitative evaluation of the role of the first nucleotide in determining the activity of the miRNAs (i.e., the differentiation-inducing activity was reduced by 77%, and the effect on cell viability was reduced by around 38% at 2 nM; Figure 2C,D). When testing the WT miR-506-3p and the three most potent differentiation-inducing analogs by colony formation assay, we found that the differentiation-inducing mimic and analogs significantly decreased the number of colonies compared to the control treatment. However, the sizes of the colonies formed by the cells that survived the differentiation-inducing treatments were not significantly reduced compared to cells treated with the control oligos. One possible explanation is the tumor heterogeneity that has been commonly observed in all types of cancers. The cells that survived miR-506-3p treatment may represent a subpopulation of cells that are resistant to miR-506-3p. However, since the colony number was significantly reduced, we could say that miR-506-3p is capable of inducing the death of a large subpopulation of neuroblastoma cells that are sensitive to miR-506-3p treatment. The heterogeneity in response to miR-506-3p treatment certainly warrants further investigation in the future.

Another important finding is that certain analogs (506-TRN5, 506-TRN6, 506-5′TRN2, and 506-TRN1-5′TRN1) slightly but significantly increased cell viability compared to the control oligos (Figure 2B,D and Figure 6), which suggests that these analogs become oncogenic to some extent. Although the molecular mechanisms behind this outcome need to be further investigated, our results indicated that these analog designs should not be further considered for potential anti-cancer agents.

In this study, we only explored deletions at limited positions in the non-seed region of miR-506-3p. It is possible that the positions that we did not investigate have distinct influences on its differentiation-inducing activity. Future investigations of these positions are certainly warranted. In addition, we and other groups have discovered other differentiation-inducing miRNAs in neuroblastoma cells [12,13,14,15,16]. Since each of these miRNAs (or seed families) has its own unique targetome, which is determined by its unique seed sequence, mimics of these miRNAs may represent distinct differentiation-regulating pathways [16]. This is especially valuable given the diverse genetic backgrounds and tumorigenesis mechanisms of neuroblastomas—a neuroblastoma case that is resistant to miR-506-3p might be sensitive to another differentiation-inducing miRNA. Shortened mimic designs of these miRNAs are certainly worth exploring in the future. These differentiation-inducing miRNAs vary in length, ranging from 20 to 23 nucleotides [16]. Although our findings with miR-506-3p can be used as preliminary guidance for designing truncated analogs of other miRNAs, the limited knowledge that we gained from this study is far from sufficient for us to simply apply our findings to other differentiation-inducing miRNA, especially miRNAs with lengths different from miR-506-3p. The activities of shortened analogs of each differentiation-inducing miRNA certainly warrant experimental validations.

## 4. Materials and Methods

### 4.1. Cell Lines and Materials

The human neuroblastoma cell lines BE(2)-C, SK-N-BE(2), and BE(2)-M17 were from the American Type Culture Collection (Manassas, VA, USA); KELLY were from the cell line repository at the Greehey Children’s Cancer Research Institute at the University of Texas Health, San Antonio. The miR-506-3p mimic, analogs, and negative control oligos were purchased from Sigma-Aldrich (St. Louis, MO, USA).

### 4.2. Reverse Transfection with miRNA and Controls

To enable the reverse transfection process in 96-well plates, 5 µL of the diluted miRNA-506-3p analogs or controls was plated into each well and incubated with 15 µL of the diluted Lipofectamine RNAiMAX for a duration of 5 min at room temperature. A total of 3000 BE(2)-C cells in 105 µL incomplete medium (DMEM/F-12 with 10% fetal bovine serum (FBS), without penicillin/streptomycin (P/S)) were then seeded into each well. The cells were then cultured in a cell incubator (37 °C, 5% CO_2_). On the next day, 80 µL of DMEM/F-12 cell culture medium supplemented with 10% FBS and 1% P/S was added to each well and the cells were cultured for another 3 days before neurite and cell viability analysis.

### 4.3. Detection and Quantification of Neurite Outgrowth

For neurite outgrowth detection, cells were imaged by a ZOOM IncuCyte Live Cell Imaging System under 20X magnification. Neurite length associated with each treatment was identified via neurite definition within the ZOOM Neurotrack program. Colorimetric representative images of the cell body and neurite lengths were then quantified.

### 4.4. MTT Assay

MTT (2-(4,5-Dimethylthiazol-2-yl)-2,5-Diphenyltetrazolium Bromide) assay, which is an assay to measure the number of viable cells based on the measurement of cellular metabolism, was conducted as previously described [36]. MTT stock solution was made at a final concentration of 2.5 mg/mL by dissolving MTT powder with sterile phosphate-buffered saline (PBS). The working MTT solution (0.25 mg/mL) was made by diluting the MTT stock solution with the full culture medium (i.e., cell culture medium supplemented with 10% FBS and 1% P/S). Cells were treated with 100 µL of MTT working solution per well and incubated at 37 °C for 2 h. After the plates were centrifuged at 2000 rpm for 5 min, the working solution was decanted and replaced with 100 µL of DMSO. Treated plates were incubated at 37 °C for 5 min. Absorbance was read with an Epoch microplate spectrophotometer at 570 nm and 630 nm. Data were recorded using the Gen5 Software interface and analyzed using Microsoft Excel and GraphPad Prism (version 7.05).

### 4.5. Colony Formation Assay

A colony formation assay was used to measure the long-term effect of treatments on cell proliferation. Briefly, cells were transfected with the respective miR-506-3p mimic or control oligos (20 nM) in 6-well plates and cultured overnight. On the second day, cells were trypsinized, and 5000 cells containing DMEM/F-12 cell culture medium supplemented with 10% FBS and 1% P/S were seeded into 100 mm dishes in three replicates. The cells were then cultured for 10–14 days or until colonies were visible. For staining the colonies, the cells were rinsed with 1X PBS and fixed/stained with 0.05% *w*/*v* crystal violet (containing 10% methanol and 1% PFA) for 20 min at room temperature. After the fixing and staining solution was decanted, colonies were washed with cold water and left overnight to air dry. Plates were scanned using a CanoScan 9000F Mark II (Canon, Tokyo, Japan). Colony numbers and sizes were quantified using ImageJ software (bundled with Java 8).

### 4.6. Statistical Analyses

To evaluate the effect of treatments, the statistical significance of the difference between any two treatment groups (e.g., between each analog and the control group) was determined by a two-tailed Student’s *t*-test, with *p* < 0.05 considered statistically significant. The IC_25_, IC_50_, and IC_75_ values of cell viability (i.e., the concentrations that reduce the cell viability by 25%, 50%, and 75%) and the EC_25_, EC_50_, and EC_75_ values of neurite outgrowth (i.e., the concentrations that lead to 25%, 50%, and 75% of the maximum/plateau neurite-inducing activity) were determined by applying the nonlinear fit variable slope (four parameters) model to the dose–response data using the Prism GraphPad software (version 7.05).

## Figures and Tables

**Table 1:** The list of 3′-end- and 5′-end-truncated miR-506-3p analogs. Shown are the (1) names of the oligonucleotides, (2) sequences of oligos with their seed sequences underlined, and (3) sequences of the carrier strands. Nucleotide deletions are indicated by dashes.

(1) Name	(2) Sequence (5′-3′)	(3) Carrier Sequence (5′-3′)
506-WT	UAAGGCACCCUUCUGAGUAGA	UACUCAGAAGGGUGCCUUAuu
506-TRN1	UAAGGCACCCUUCUGAGUAG-	-ACUCAGAAGGGUGCCUUAuu
506-TRN2	UAAGGCACCCUUCUGAGUA--	--CUCAGAAGGGUGCCUUAuu
506-TRN3	UAAGGCACCCUUCUGAGU---	---UCAGAAGGGUGCCUUAuu
506-TRN4	UAAGGCACCCUUCUGAG----	----CAGAAGGGUGCCUUAuu
506-TRN5	UAAGGCACCCUUCUGA-----	-----AGAAGGGUGCCUUAuu
506-TRN6	UAAGGCACCCUUCUG------	------GAAGGGUGCCUUAuu
506-5’TRN1	-AAGGCACCCUUCUGAGUAGA	UACUCAGAAGGGUGCCUU-uu
506-5’TRN2	--AGGCACCCUUCUGAGUAGA	UACUCAGAAGGGUGCCU--uu
506-TRN1-5’TRN1	-AAGGCACCCUUCUGAGUAG-	-ACUCAGAAGGGUGCCUU-uu

**Table 2:** The list of shortened miR-506-3p analogs with nucleotide deletions in the middle positions of the miR-506-3p sequence. Shown are the (1) names of the analogs, (2) sequences of the analogs, and (3) sequences of the carrier strands.

(1) Name	(2) Sequence (5′-3′)	(3) Carrier Sequence (5′-3′)
506-Mid#13	UAAGGCACCCUU-UGAGUAGA	UACUCA-AAGGGUGCCUUAuu
506-Mid#13-14	UAAGGCACCCUU--GAGUAGA	UACUC--AAGGGUGCCUUAuu
506-Mid#13-15	UAAGGCACCCUU---AGUAGA	UACU---AAGGGUGCCUUAuu
506-Mid#13-16	UAAGGCACCCUU----GUAGA	UAC----AAGGGUGCCUUAuu
506-Mid#15	UAAGGCACCCUUCU-AGUAGA	UACU-AGAAGGGUGCCUUAuu
506-Mid#16	UAAGGCACCCUUCUG-GUAGA	UAC-CAGAAGGGUGCCUUAuu
506-Mid#15-16	UAAGGCACCCUUCU--GUAGA	UAC--AGAAGGGUGCCUUAuu

**Table 3:** The list of miR-506-3p analogs made by combining deletions at the 3′ end, deletions in the middle positions, and additions of 5′ U. Shown are the (1) names of the analogs, (2) sequences of the analogs, and (3) sequences of the carrier strands.

(1) Name	(2) Sequence (5′-3′)	(3) Carrier Sequence (5′-3′)
TRN2-Mid #13	UAAGGCACCCUU-UGAGUA--	--CUCA-AAGGGUGCCUUAuu
TRN2-Mid #15	UAAGGCACCCUUCU-AGUA--	--CU-AGAAGGGUGCCUUAuu
TRN2-Mid#13,15	UAAGGCACCCUU-U-AGUA--	--CU-A-AAGGGUGCCUUAuu
U+TRN3	UUAAGGCACCCUUCUGAGU---	---UCAGAAGGGUGCCUUAAuu
U+TRN2-Mid#13,15	UUAAGGCACCCUU-U-AGUA--	--CU-A-AAGGGUGCCUUAAuu

## Data Availability

Not applicable.

## Sample Availability

Not available.
